# Supplementary material for: Perivascular Stromal Cells Instruct Glioblastoma Invasion, Proliferation, and Therapeutic Response within an Engineered Brain Perivascular Niche Model
Source: Adv Sci (Weinh). 2022 Sep 15;9(31):2201888. doi: 10.1002/advs.202201888 (PMC9631060; doi:10.1002/advs.202201888)
Supplement: Supplementary file 1 — Supporting information [file ADVS-9-2201888-s001.pdf]

Supporting Information

Perivascular Stromal Cells Instruct Glioblastoma Invasion, Proliferation, and Therapeutic Response within an Engineered Brain Perivascular Niche Model

*Mai T. Ngo, Jann N. Sarkaria, Brendan A.C. Harley**

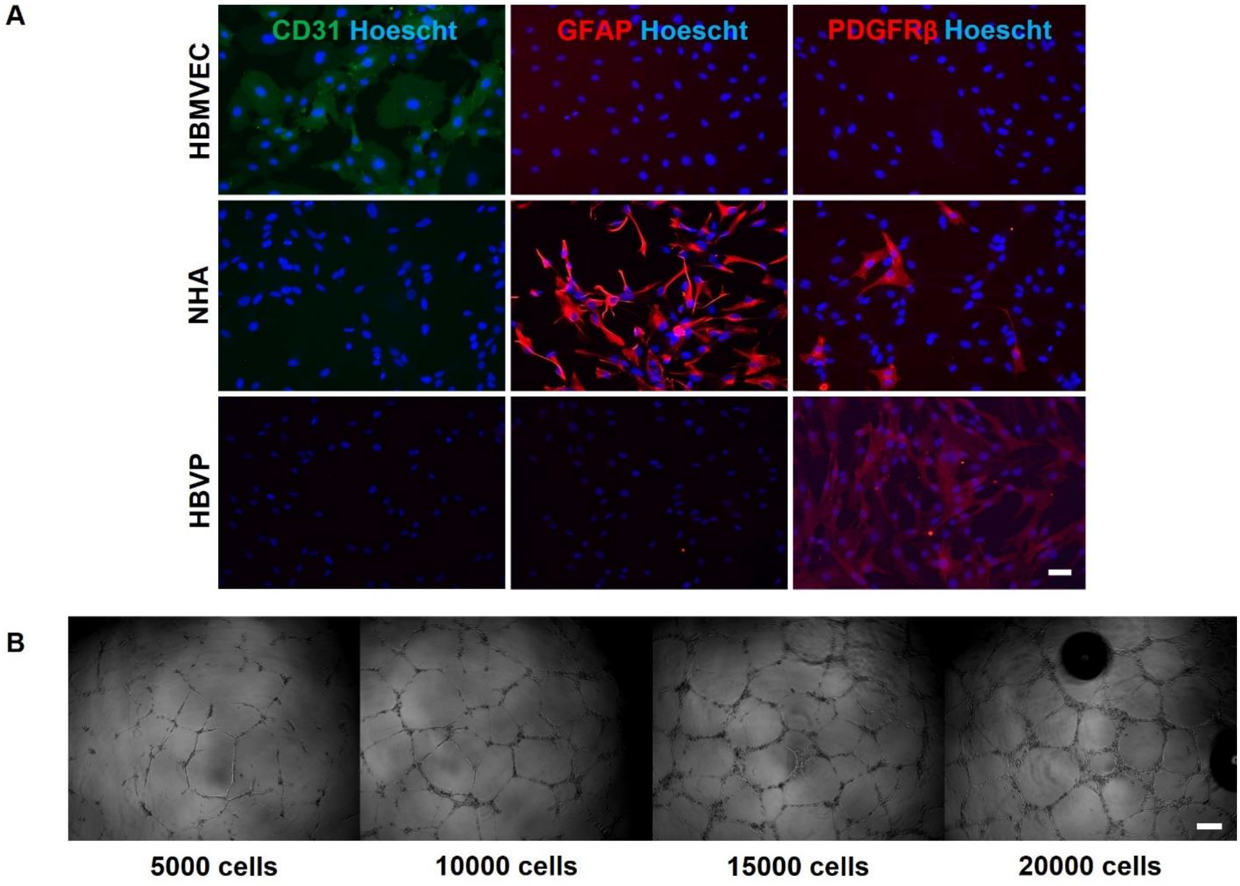

Figure S1. (A) Immunofluorescent markers for human brain microvascular endothelial cells (HBMVEC), normal human astrocytes (NHA), and human brain vascular pericytes (HBVP). Cells were fixed 48 hours after seeding. Scale bar = 50 μm. **(B)** Matrigel tube formation assay for brain microvascular endothelial cells. Images were taken 16 hours after cell seeding. Scale bar = 100 μm

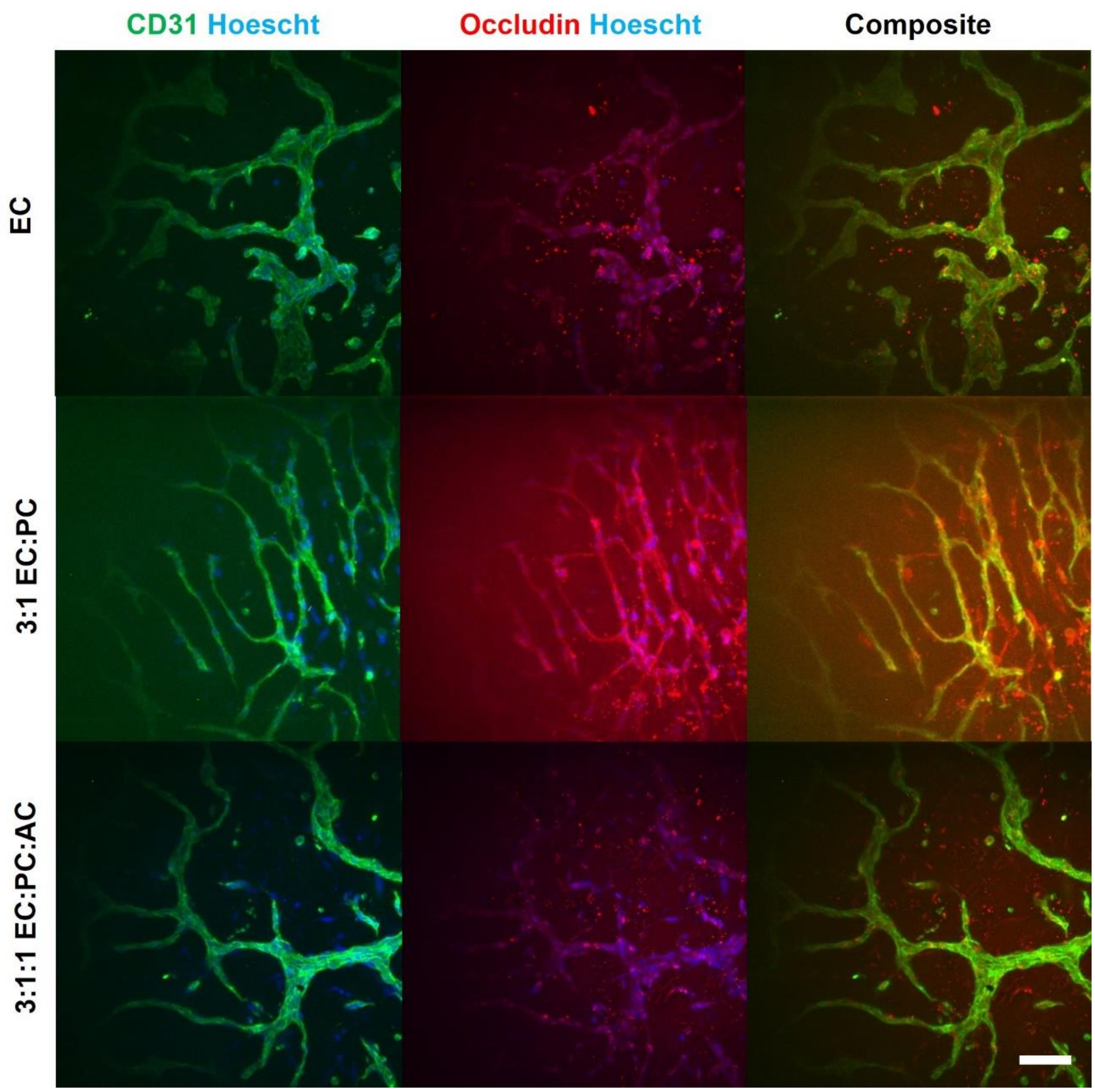

Figure S2. Immunofluorescent staining for occludins in microvascular cultures. Scale bar = 100 μm.

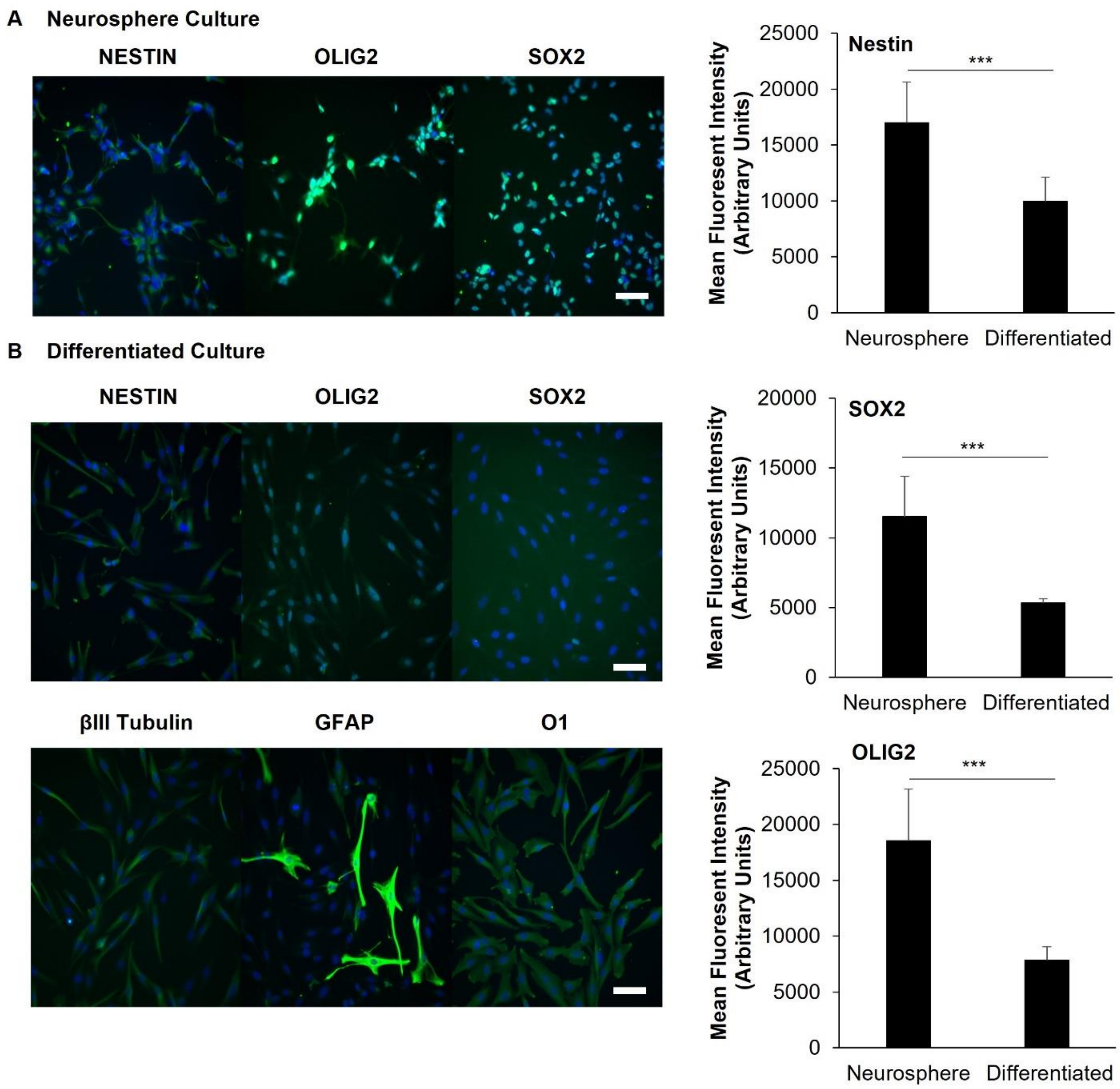

Figure S3. (A) Immunofluorescent staining for stem markers in GBM6 cells that have been grown in neurosphere culture and plated on laminin. Scale bar = 100 um. **(B)** Immunofluorescent staining for stem and differentiated markers for GBM6 cells grown in media containing FBS for two weeks. Scale bar = 100 μm. **(C)** Quantification of stem marker expression (assessed via mean fluorescent intensity) between neurosphere and differentiated cultures. Data analyzed using Mann-Whitney tests; ***p<0.001.

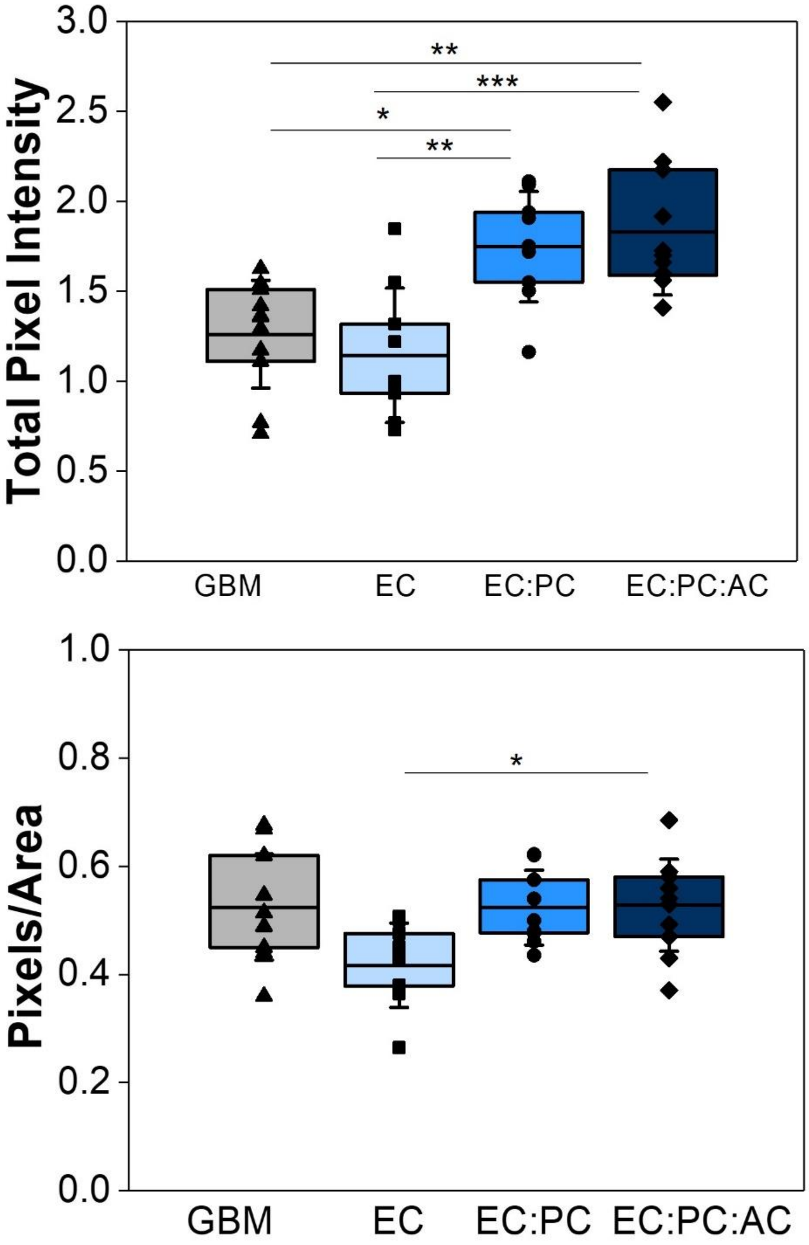

Figure S4. Quantification of total pixel intensity and total pixel intensity normalized to outgrowth area in spheroid invasion cultures. Data analyzed using one-way ANOVA with Scheffe's post-hoc; *$p<0.05$, **$p<0.01$, ***$p<0.001$, N = 9 – 11 hydrogels.

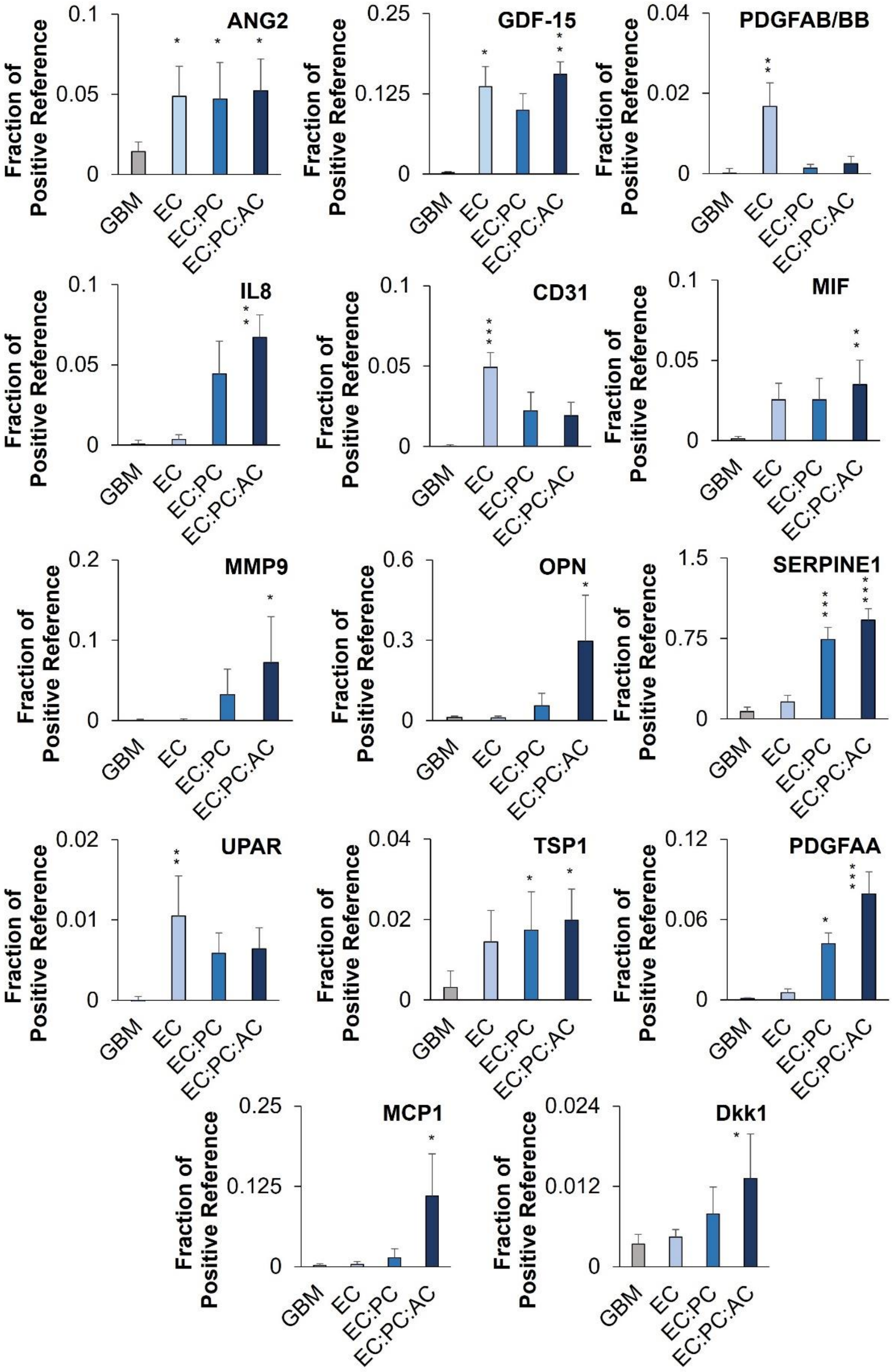

Figure S5. Quantitative comparison of proteins secreted by spheroid invasion cultures. Data analyzed using one-way ANOVA with Tukey's post-hoc or Kruskal-Wallis test with Dunn's post-hoc; *$p<0.05$, **$p<0.01$, ***$p<0.001$ compared to GBM6-only cultures; N = 5 conditioned media samples.

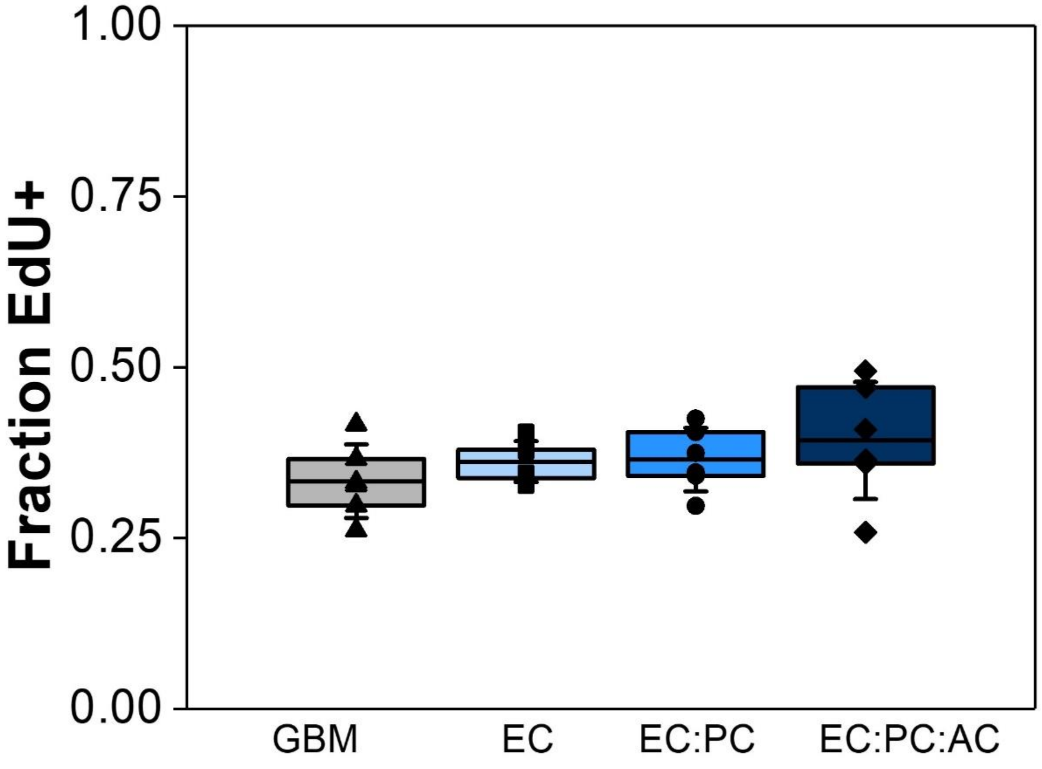

Figure S6. Quantification of the fraction of RFP-expressing GBM6 tumor cells that incorporate EdU in a 24-hour pulse. N = 6 hydrogels.

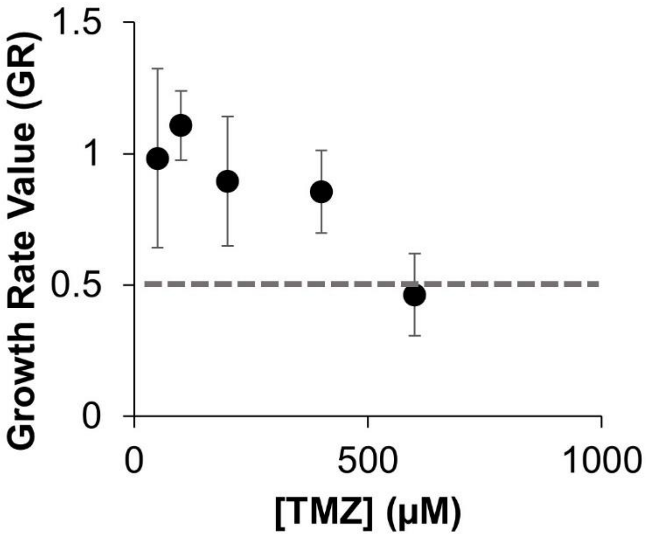

Figure S7. Dose-response curve for GBM12 cells treated with temozolomide for 48 hours. N = 4 - 6 hydrogels.

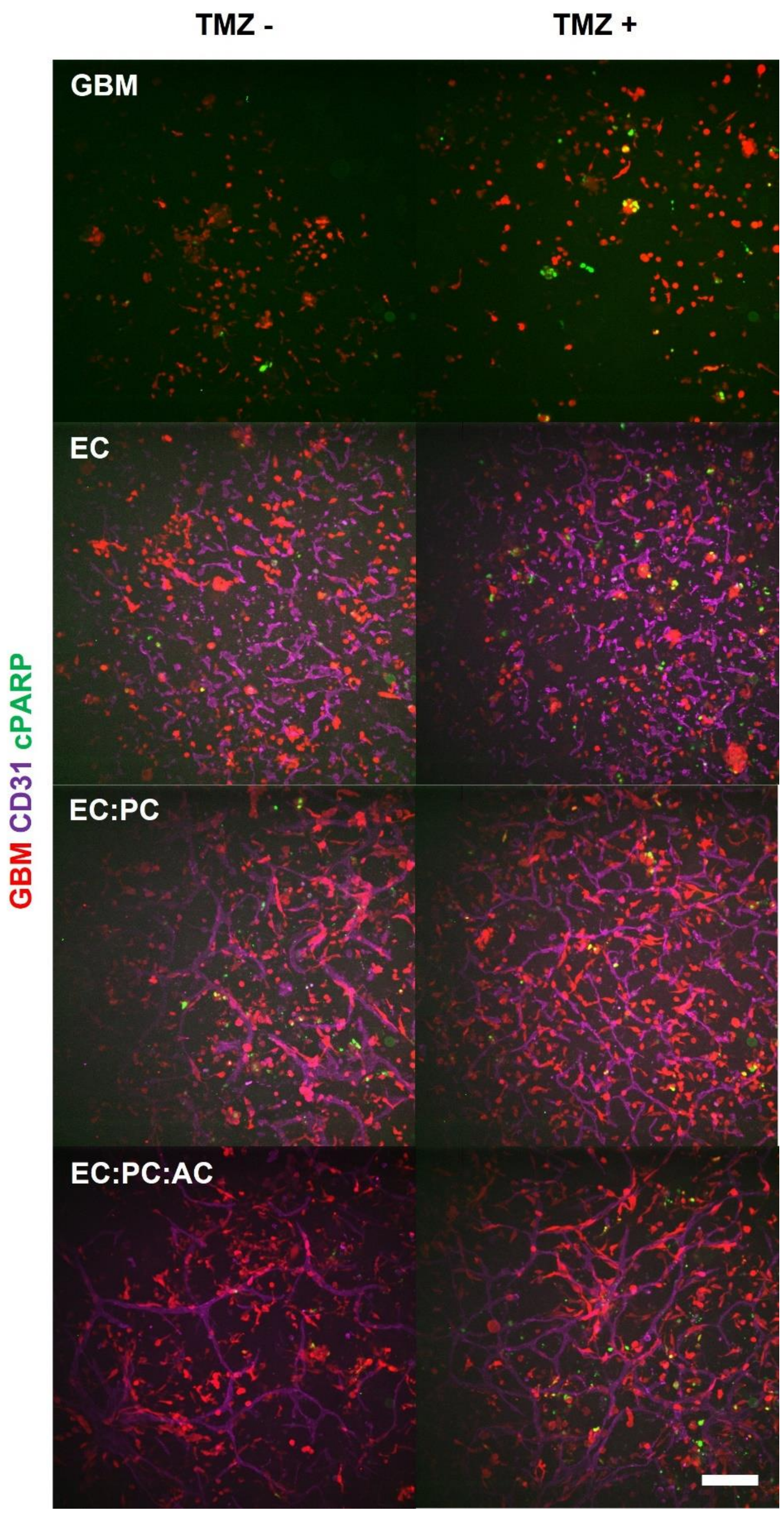

Figure S8. Representative images of tumor-vascular co-cultures with and without 48 hours of temozolomide treatment. cPARP staining was performed at the end of the treatment period. Scale bar = 200 μm.

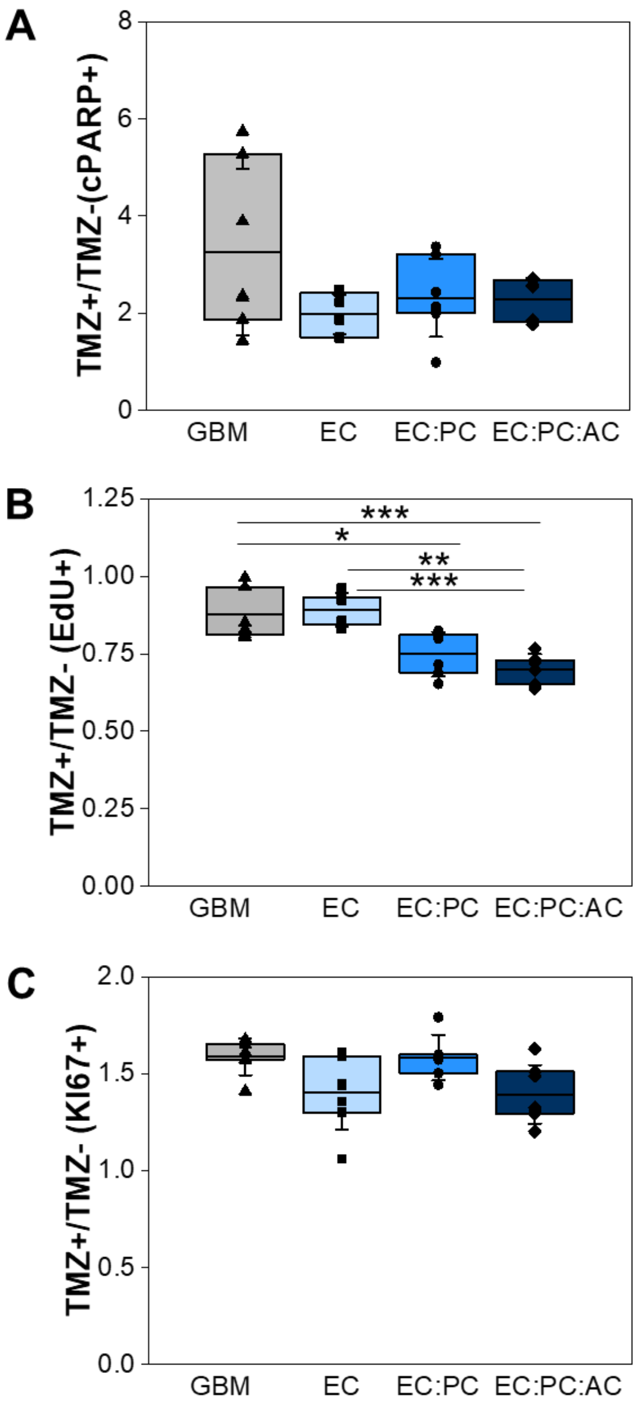

Figure S9. Calculated ratios for **A)** cPARP**, B)** EdU**,** and **C)** KI67 expression between TMZ-treated (TMZ+) and DMSO control (TMZ-) groups. * $p<0.05$, **$p<0.01$, and ***$p<0.001$ between groups.

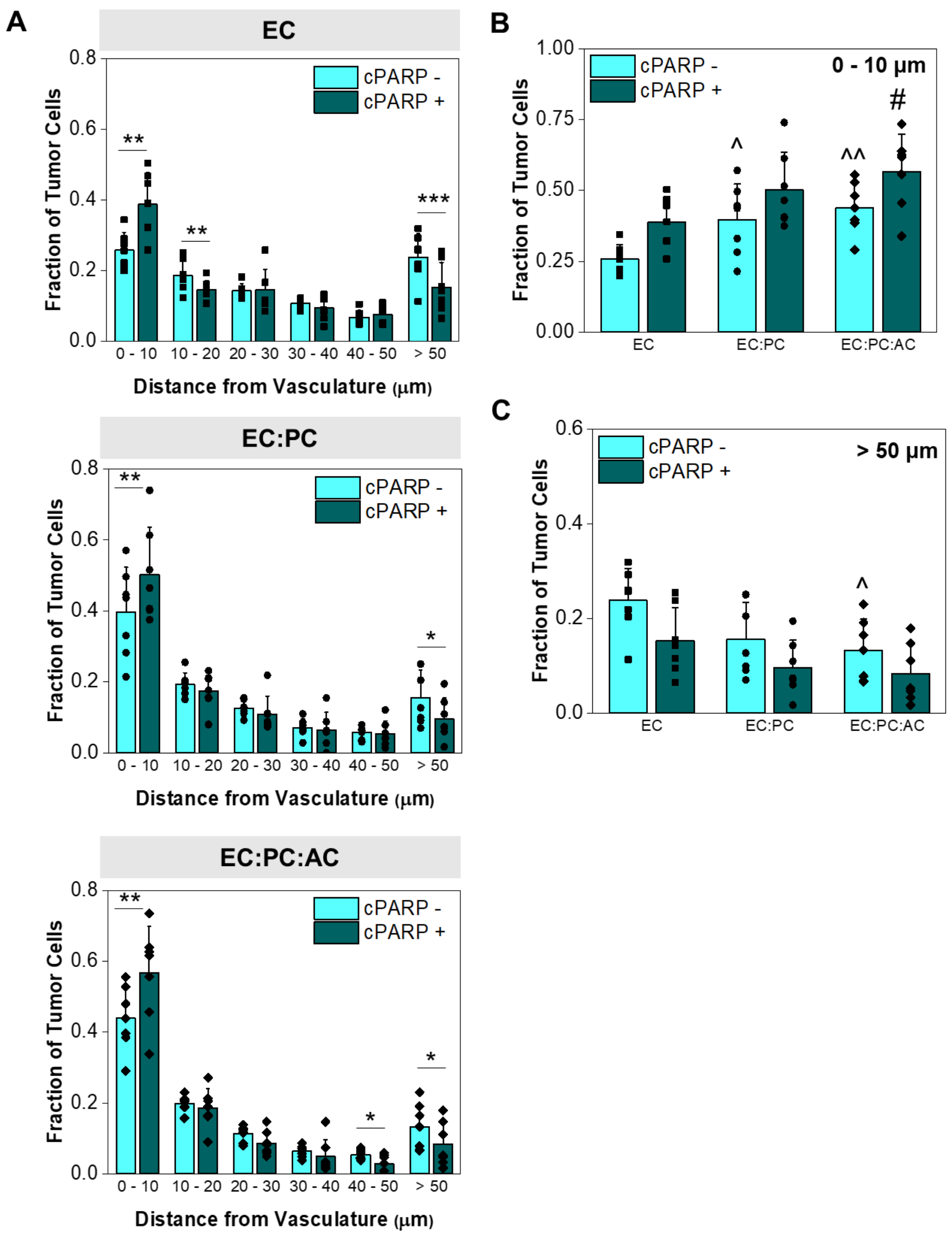

Figure S10. A) Comparing the proximity of cPARP- vs. cPARP+ GBM12 cells to the microvascular network after temozolomide treatment. Data points represent the fraction of tumor cells that are a specified distance from the microvascular network in a single hydrogel. Data analyzed using paired-sample t-test or Wilcoxon signed-rank test;*p<0.05, **p<0.01, ***p<0.001 **B)** Comparing the fraction of cPARP- and cPARP+ GBM12 cells that are within 10 µm of the microvascular network. Data analyzed using one-way ANOVA with Tukey's post-hoc; ^p<0.05, ^^p<0.01 compared to EC, cPARP-; #p>0.05 compared to EC, cPARP+ **C)** Comparing the fraction of cPARP- and cPARP+ GBM12 cells that are greater than 50 µm from the microvascular network. Data analyzed using one-way ANOVA with Tukey's post-hoc; #p<0.05 compared to EC, cPARP-; N = 7 hydrogels

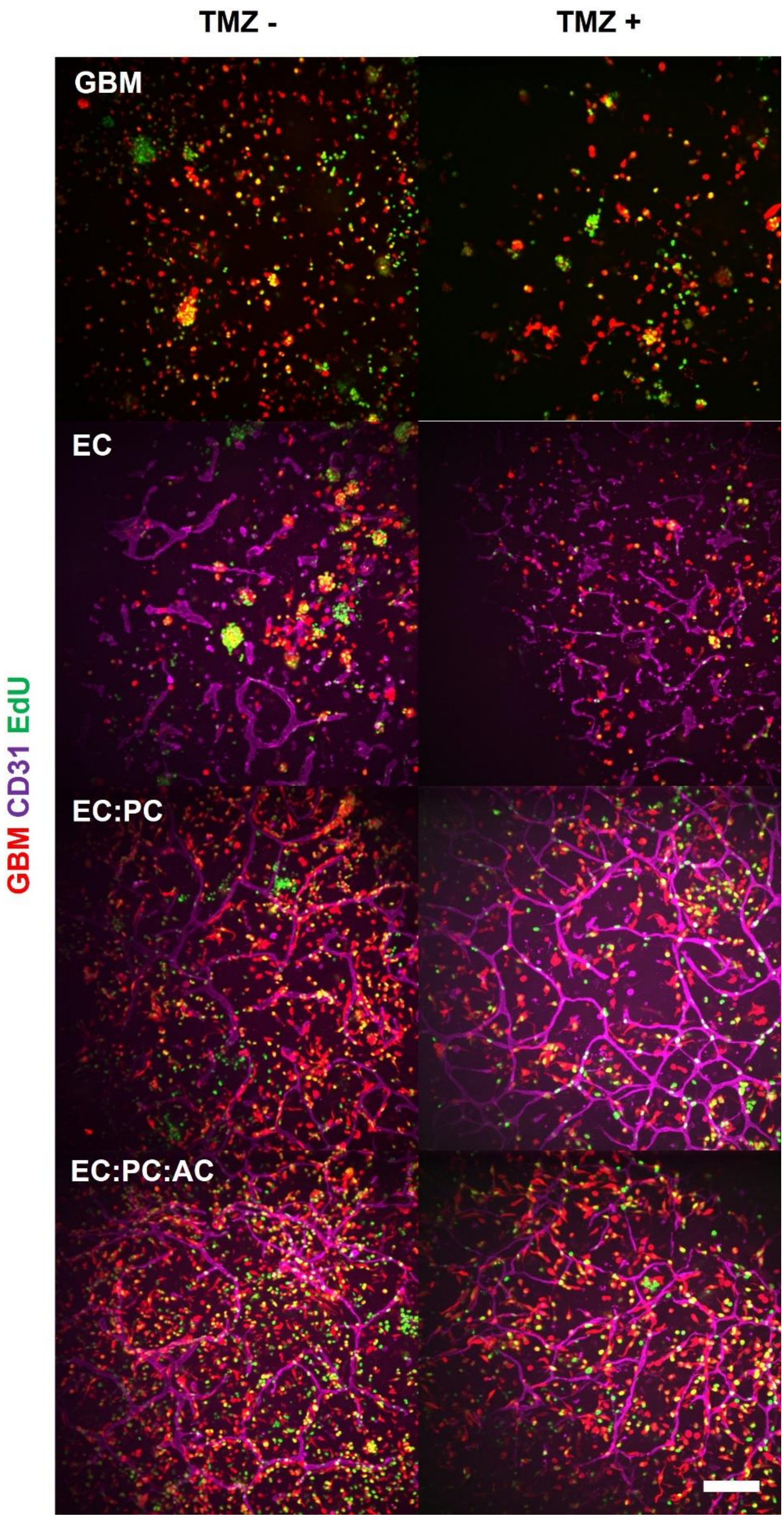

Figure S11. Representative images of tumor-vascular co-cultures with and without 48 hours of temozolomide treatment. EdU staining occurred after a 24-hour pulse of EdU after temozolomide treatment. Scale bar = 200 μm.

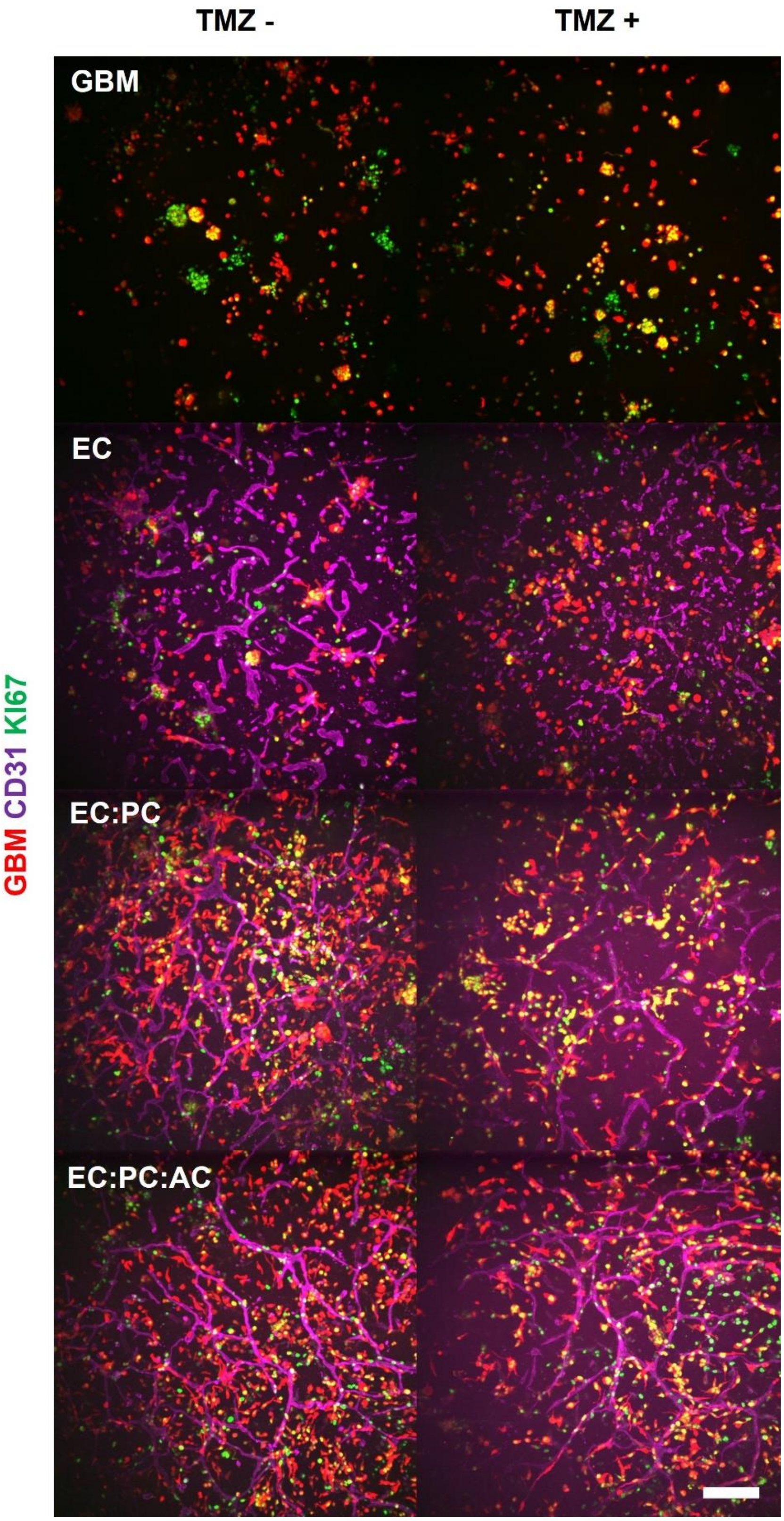

Figure S12. Representative images of tumor-vascular co-cultures with and without 48 hours of temozolomide treatment. KI67 staining was performed at the end of the treatment period. Scale bar = 200 µm.
